# Infants’ interoception is associated with eye contact in dyadic social interactions

**DOI:** 10.1038/s41598-023-35851-9

**Published:** 2023-06-19

**Authors:** Masahiro Imafuku, Hiromasa Yoshimoto, Kazuo Hiraki

**Affiliations:** 1 Department of Early Childhood Education and Care, Faculty of Education, Musashino University, 1-1-20 Shin-Machi, Nishitokyo-Shi, Tokyo, 202-8585 Japan; 2 Graduate School of Arts and Sciences, The University of Tokyo, 3-8-1 Komaba, Meguro-Ku, Tokyo, 153-8902 Japan

**Keywords:** Human behaviour, Social behaviour

## Abstract

Interoception, that is, the perception of visceral stimuli, is the basis of socio-emotional development. However, no studies have demonstrated the relationship between the two in infants. This study aimed to elucidate the relationship between interoception and social behavior in infants and mothers. Visual preference for cardio-visual synchronous and asynchronous stimuli was assessed using a preferential-looking paradigm in 6-month infants and their mothers. The infant–mother interaction was also measured to assess social behavior, such as eye contact and positive facial expressions. The results showed that infants looked at asynchronous cardio-visual stimuli longer than synchronous cardio-visual stimuli, whereas mothers looked at synchronous cardio-visual stimuli longer than asynchronous cardio-visual stimuli. The proportion of looking time toward asynchronous cardio-visual stimuli in infants was positively correlated with infant–mother gaze and affect (positive facial expression) synchrony. Furthermore, mediation analyses showed that the relationship between infants’ interoception and eye contact behavior is attributable to mother’s positive facial expression. Our findings suggest that in infant–mother interactions, infants’ interoception may play a role in eye contact behavior through the mother's positive facial expression, highlighting the importance of infants’ interoception on social cognitive development.

## Introduction

Interoception, the sense of the physiological state of the body, is thought to be critical for the subjective feeling of emotion and self-consciousness^1–3^. Recent studies have revealed the role of interoception in social cognition, such as sensitivity to social cues (e.g., stimuli that indicate relevant information in an interpersonal context, including others’ faces, gaze, voice, or touch), imitation, and empathy in adults^4–9^, although a previous study has found no association^10^. However, little is known about the association between interoception and social cognition during infancy.

A recent hypothesis suggests that interoception is the basis for social cognitive development^11–13^. According to this line of thinking, the perception of interoceptive information, such as cardiac signals from the body, is associated with the perception of exteroceptive information, such as that from caregivers and surrounding adults. For example, when infants are held by their parents and parents smile at them, they feel warmth and security through their interoceptive signals, and feel attached to their parents. Through the experience of interacting with others, the internal model of the caregiver is acquired as a reward, which promotes infant attachment and motivation for social interaction^13^. Therefore, it is assumed that infants who are more sensitive to interoception are more likely to give more weight to exteroception, which may be associated with sensitivity to social cues.

Interoception has been investigated using behavioral measures in 5-month-old infants^14^. In this study, the infant's looking time was measured for each figure that moved synchronously with the infant's heartbeat and asynchronously with it, presented side-by-side on the display. The results showed that infants looked at the figure moved asynchronously with their heartbeat longer than the figure moved synchronously with it, suggesting that infants by 5 months of age had the ability to detect cardio-visual (interoception–exteroception) synchrony. While there is evidence that infants can detect interoceptive signals, it remains to be determined whether individual differences in the degree to which interoceptive signals are detected are associated with differences in social behavior during infancy.

Social cognition develops through social interactions^15^. Infants synchronize their eye gaze, vocalizations, affect, and touch with their parents in their daily social interactions (for a review, see Leclère et al*.*^16^). Infant–parent synchrony has been shown to facilitate cognitive, socio-emotional, and self-regulatory development (e.g., Feldman^17^). In social interactions, infants require the ability to detect social cues^16^. If interoception is related to sensitivity to socially relevant cues^8,9^, in mother-infant interactions, it may function under the influence of the social behavior of others, such as eye contact and smiling, leading to the social behavior of the infant. As interoception is thought to be involved in the acquisition of an internal model in which caregivers (or others around them) are the reward^13^, infants with high sensitivity to interoception are likely to be more motivated to engage with others. This process is considered to facilitate learning about social cues, and thus, social cognitive development. Therefore, it is possible that infants’ interoception may be related to social behaviors in social interactions; however, no studies have examined this evidence, to the best of our knowledge.

Interoception can also be a key component of parenting^18,19^. Parents who are accurately aware of sensations inside the body and their own emotions are thought to be better able to notice their children's emotional states. It is possible that parents with high interception are more likely to engage in social behavior with their children, and thus, their children may be more likely to develop social cognitive skills. For example, mothers’ interoceptive knowledge of emotions was associated with approximately 8 years of children’s social cognitive skills such as emotion regulation and self-control^19^. What remains to be determined is the relationship between mother’s interoception and infant’s social behaviors.

The current study aimed to elucidate the role of infants’ interoception in social behavior in mother–infant interactions by investigating the relationship between interoception and social behaviors such as eye contact and smiling in infants and mothers. To do so, we assessed the interoception of infants and mothers by measuring the time spent looking at figures that move synchronously or asynchronously with their own heartbeats. We also assessed social behaviors such as eye contact and smiling during mother–infant interactions. First, we predicted that infants would indicate a preference for asynchronous cardio-visual stimuli because 6-month-olds are thought to experience the sensation of heartbeats in daily life^14^, they may regard synchronous cardio-visual stimuli as familiar and show novelty preference (preference for asynchronous cardio-visual stimuli)^20^. Although children up to the age of 4 years tend to prefer novel stimuli, adults tend to prefer familiar stimuli^21,22^. Therefore, mothers would indicate a preference for synchronous cardio-visual stimuli. Second, if interoception is associated with social behaviors toward others^13^ and parenting behavior^18,19^, we predicted that infants’ preference for asynchronous cardio-visual stimuli would be positively correlated with the degree of social behaviors toward mothers, whereas mothers’ preference for synchronous cardio-visual stimuli would be positively correlated with the degree of social behaviors toward infants. Third, if infants' interoception functions through infant–mother interaction, leading to their social behavior, then infants with higher sensitivity to interoception would be more socially engaged by their mothers and consequently engage in more social behavior.

## Methods

### Participants

Participants were 27 monolingual Japanese infants aged 6 months (*M* = 200.26 days, *SD* = 16.89) and their mothers (*M* = 34.19 years, *SD* = 4.49). None of the participants reported a history of psychiatric or neurological conditions. Parents provided written informed consent for this study, which was approved by the Ethics Committee of the University of Tokyo, and was therefore conducted in accordance with the standards specified in the 1964 Declaration of Helsinki. Data from two infants and three mothers were excluded from the analysis of the cardio-visual preferential looking task because the infants were not able to conduct the study due to fussiness, one mother was unable to conduct the study due to equipment problems that prevented detection of heartbeats, and two mothers were not due to the fussiness of their infants.

### General procedure

Parents provided written informed consent for this study. All methods were approved by the Ethics Committee of the University of Tokyo, and was therefore conducted in accordance with the standards specified in the 1964 Declaration of Helsinki.

### Cardio-visual preferential looking task

The heartbeat of mothers and infants was measured using an electromyography (EMG) biofeedback device^23^. Measurements were obtained by attaching three EMG leads to the infant's chest. The mothers were given prior instructions on the placement of the leads and subsequently attached them to their own chests. The cardiac R wave of the heartbeat was extracted, and a square figure (blue or orange) that moved up and down in time with the peak of the R wave (cardio-visual synchronous stimulus) and a square figure (orange or blue) that moved up and down asynchronously with the peak of the R wave (cardio-visual asynchronous stimulus) were presented in pairs on the left and right sides of the display, respectively. The cardio-visual synchronous stimulus was controlled to move at the same time as the R wave, and the cardio-visual asynchronous stimulus was controlled to move at a time that was 10% faster or slower than the heartbeat interval between the previous and latest R waves. The extraction of the R wave of the heartbeat and control of the stimulus were performed using a Python script.

**Figure 1:**
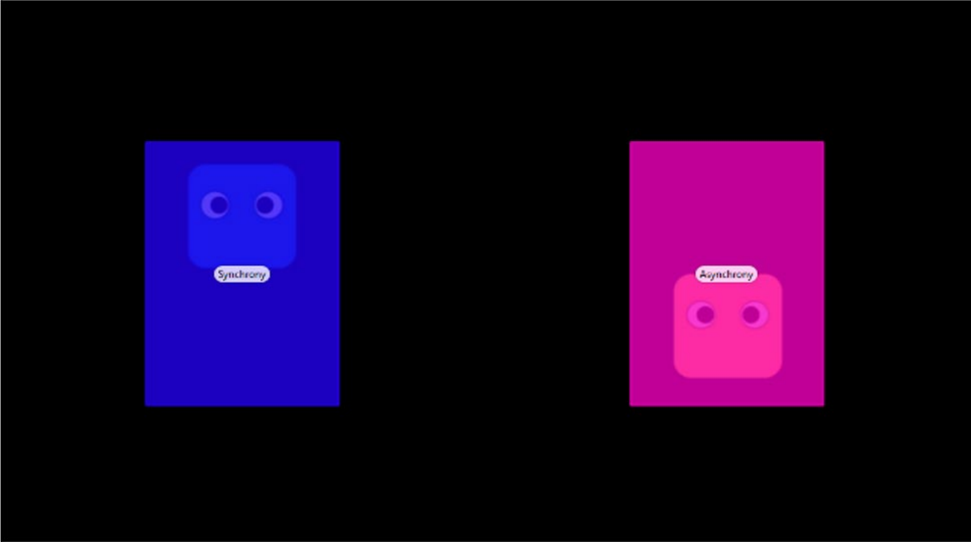
Examples of movie stimuli and areas of interest.

We presented a cardio-visual synchronous and asynchronous stimulus on a 23-inch wide color monitor (screen resolution 1920 × 1080 pixels) and recorded participants’ eye movements using an infrared bright pupil eye-tracking system (Tobii TX300, Tobii Technology AB). The system recorded near-infrared reflections of both eyes at 300 Hz with an accuracy of 0.5°. Stimulus presentation and recording were controlled using the Tobii Studio software (Tobii Technology AB). The areas of interest (AOIs) consisted of the left and right areas, which covered the cardio-visual synchronous and asynchronous stimuli (Fig. 1).

There were four trials, each lasting 40 s. The cardio-visual synchronous and asynchronous stimuli were presented in alternating positions (left and right), and the location of the presentation was counterbalanced across participants. As for the asynchronous stimuli, two trials for the faster and two trials for the slower asynchronous condition were presented alternately, and the order of presentation was counterbalanced across the participants. We calculated the proportion of looking time (PLT) toward the cardio-visual synchronous stimuli by dividing the total fixation time the participants spent looking at the synchronous stimuli by the total fixation time the participants looked at the synchronous and incongruent stimuli, and the PLT toward the cardio-visual asynchronous stimuli by dividing the total fixation time the participants spent looking at the asynchronous stimuli by the total fixation time the participants looked at the synchronous and asynchronous stimuli.

### Social behaviors in infants and mothers

Mothers were instructed to play freely with the infant for 3 min without using things. Two video cameras were set up to capture the face-to-face play of the mother and infant so that the entire face and body of the mother and infant were captured. During this time, the experimenter moved out of the room so that only the infant and mother were in the room.

Infants and mothers’ behaviors were coded offline by two trained individuals (coders) at 1000 ms intervals using a frame-by-frame advance. We calculated the frequency of infants and mothers’ social behaviors (eye gaze, vocalizations, emotional expressions, and touch). Infants’ eye gaze was their gaze behaviors to mother’s face. Infants’ positive vocalizations were babbling or cooing, and their negative vocalizations were fussiness or crying. Infants’ emotional expressions were categorized into three categories (positive facial expression (smiling or laughing), negative facial expression (sad or fussy), and neutral facial expression. The infant’s touch was the action of the infant touching the mother's body with its hands. The mother’s gaze was their gaze behaviors to the infant’s face. Mothers’ vocalizations were categorized into three categories (infant-directed speech [positive vocalization; high-pitched speech or speech, including singing], adult-directed speech and low- or middle-pitched speech). Mothers’ emotional expressions were categorized into three categories (positive facial expression (smiling or laughing), negative facial expression (sad or fussy), and neutral facial expression. Mothers’ touch was defined as the action of a mother touching the infant's body with her hands. We also computed four forms of behavioral synchrony^24^: (1) gaze synchrony—the proportion of time infants and mothers were in eye gaze and looked at each other; (2) vocal synchrony—the proportion of time infants and mothers emitted positive vocalizations simultaneously; (3) affect synchrony—the proportion of time infants and mothers matched their positive facial expression; and (4) touch synchrony—the proportion of time infants and mothers touched each other’s bodies.

To check the reliability of the first coder, a second coder, who had no knowledge of the aim of the experiment, coded 40% of the data (10 out of 27 pairs). The inter-coder reliability was sufficiently high (Cohens’ kappa (*κ*), *κ* > 0.85; see Appendix A for details). Thus, we used behavioral data coded by the first observer for analysis.

### Data analysis

We conducted a paired *t*-test (two-tailed) to determine whether the proportion of looking time (PLT) toward the cardio-visual synchronous stimuli was significantly different from that toward the cardio-visual asynchronous stimuli in the infant and mother groups, respectively. Data were included if the total time a participant spent looking at the two AOIs was more than 20 s (one-eighth) of 160 s. Based on these criteria, 25 infants and 24 mothers were included in the analysis of the cardio-visual preferential looking task. We then calculated Pearson's correlations between preference for cardio-visual stimuli and social behaviors to investigate whether the integration of interoception and exteroception is associated with social behaviors in infant–mother interactions. Furthermore, we conducted a mediation analysis to investigate the role of interoception in social behaviors. When parametric assumptions were not met with the data, we used angular transformation.

## Results

The mean value for infants’ PLT toward the cardio-visual synchronous stimuli was 0.455 (*SD* = 0.061), and that toward the cardio-visual asynchronous stimuli was 0.545 (*SD* = 0.061) (PLT toward the − 10% (slower) cardio-visual asynchronous stimuli was 0.559 (*SD* = 0.075), and PLT toward + 10% (faster) cardio-visual asynchronous stimuli was 0.536 (*SD* = 0.091). The mean value of the mother's PLT toward the cardio-visual synchronous stimuli was 0.542 (*SD* = 0.093), that toward the cardio-visual asynchronous stimuli was 0.458 (*SD* = 0.093) (that toward the − 10% (slower) cardio-visual asynchronous stimuli was 0.452 (*SD* = 0.098), and that toward + 10% (faster) cardio-visual asynchronous stimuli was 0.463 (*SD* = 0.112). The mean values and standard deviations for infants’ gazes towards their mothers’ faces, positive facial expressions, positive vocalization, and touch were 0.296 (*SD* = 0.227), 0.289 (*SD* = 0.232), 0.057 (*SD* = 0.060), and 0.475 (*SD* = 0.411), respectively. The mean values and standard deviations for mothers’ gazes towards the infants’ faces, positive facial expressions, positive vocalization, and touch were 0.834 (*SD* = 0.208), 0.574 (*SD* = 0.283), 0.515 (*SD* = 0.253), and 0.904 (*SD* = 0.165), respectively. The mean values and standard deviations for gaze synchrony, vocal synchrony, affect synchrony, and touch synchrony were 0.267 (*SD* = 0.216), 0.027 (*SD* = 0.029), 0.241 (*SD* = 0.223), and 0.464 (*SD* = 0.405), respectively.

### Preference for cardio-visual stimuli

**Figure 2:**
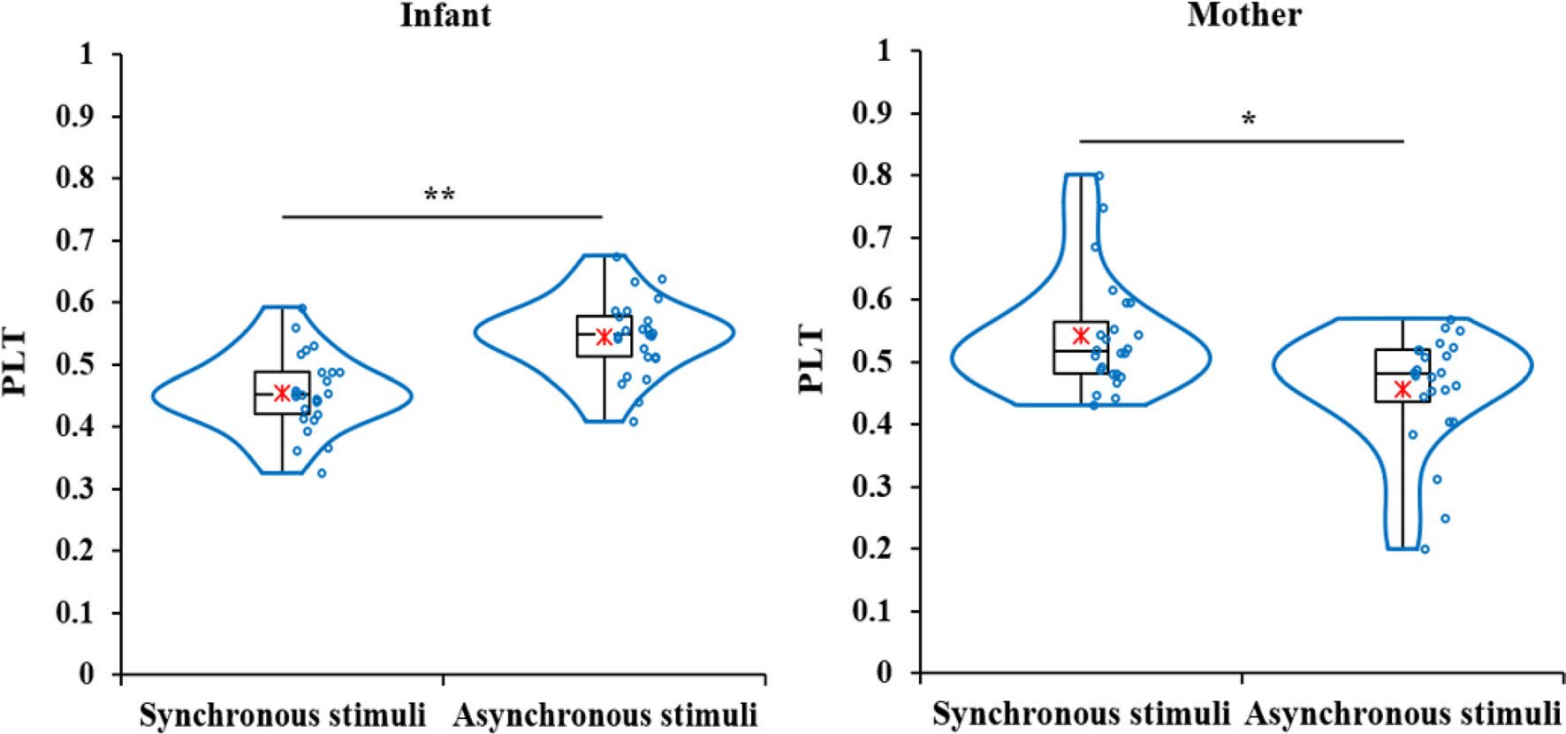
Box and Violin plots for PLT toward cardio-visual synchronous and asynchronous stimuli in infant (*N* = 25; left) and mother (*N* = 24; right). **p < 0.01, *p < 0.05.

A paired *t*-test confirmed that the PLT toward asynchronous cardio-visual stimuli was significantly higher than that synchronous cardio-visual stimuli in infants, *t*(24) = − 3.65, *p* = 0.001, *d* = − 1.47, and the PLT toward synchronous cardio-visual stimuli was significantly higher than that asynchronous cardio-visual stimuli in mothers, *t*(23) = 2.23, *p* = 0.036, *d* = 0.91 (Fig. 2).

### Relationships between preference for cardio-visual stimuli and social behaviors

The relationships between the variables were tested using Pearson's correlations, as shown in Table 1. The PLT toward the cardio-visual asynchronous stimuli in infants was positively correlated with infants’ eye gaze toward the mother’s face, mother’s positive facial expression, gaze synchrony, and affect synchrony (*r*(23) = 0.51, *p* = 0.009, 95% CI [0.149–0.756]).; *r*(23) = 0.59, *p* = 0.002, 95% CI [0.248–0.796]; *r*(23) = 0.52, *p* = 0.008, 95% CI [0.156–0.759];* r*(23) = 0.49, *p* = 0.014, 95% CI [0.112–0.739]). PLT toward the cardio-visual synchronous stimuli in mothers was positively correlated with infants’ eye gaze toward the mother’s face and infants’ positive vocalization (*r*(22) = 0.41, *p* = 0.044, 95% CI [0.013–0.700]; *r*(22) = 0.43, *p* = 0.038, 95% CI [0.026–0.707]).

**Table 1:** Correlation among variables.

	1	2	3	4	5	6	7	8	9	10	11	12	13	14	15	16
1. Infant's PLT to synchronous stimuli	–															
2. Infant's PLT to asynchronous stimuli	− 1.000	–														
3. Mother's PLT to synchronous stimuli	0.271	− 0.271	–													
4. Mother's PLT to asynchronous stimuli	− 0.271	0.271	− 1.000	–												
5. Infant's eye gaze	− 0.514**	0.514**	0.414*	− 0.414*	–											
6. Infant's positive facial expression	− 0.256	0.256	0.180	− 0.180	0.330^+^	–										
7. Infant's positive vocalization	0.068	− 0.068	0.425*	− 0.425*	0.151	0.318	–									
8. Infant's touch	0.201	− 0.201	− 0.185	0.185	− 0.075	− 0.301	− 0.245	–								
9. Mother's eye gaze	− 0.237	0.237	0.040	− 0.040	0.325^+^	0.522**	− 0.045	0.081	–							
10. Mother's positive facial expression	− 0.586**	0.586**	0.122	− 0.122	0.657**	0.312	0.238	− 0.020	0.273	–						
11. Mother's positive vocalization	0.255	− 0.255	− 0.217	0.217	− 0.324^+^	− 0.181	− 0.047	− 0.282	− 0.407*	− 0.306	–					
12. Mother's touch	0.362^+^	− 0.362^+^	0.161	− 0.161	− 0.179	− 0.005	0.348^+^	0.152	− 0.305	− 0.073	0.069	–				
13. Gaze synchrony	− 0.519**	0.519**	0.393^+^	− 0.393^+^	0.984**	0.391*	0.151	− 0.087	0.444*	0.637**	− 0.301	− 0.199	–			
14. Vocal synchrony	− 0.005	0.005	0.251	− 0.251	− 0.046	0.308	0.725**	− 0.478*	0.046	0.083	0.138	0.150	− 0.002	–		
15. Affect synchrony	− 0.485*	0.485*	0.123	− 0.123	0.446*	0.874**	0.386*	− 0.388*	0.466*	0.552**	− 0.155	− 0.061	0.497**	0.392*	–	
16. Touch synchrony	0.232	− 0.232	− 0.176	0.176	− 0.093	− 0.297	− 0.197	0.992**	0.065	− 0.033	− 0.272	0.195	− 0.104	− 0.445*	− 0.387*	–

Note: ^+^*p* < 0.10, *p < 0.05, **p < 0.01.

### Mediation analysis

**Figure 3:**
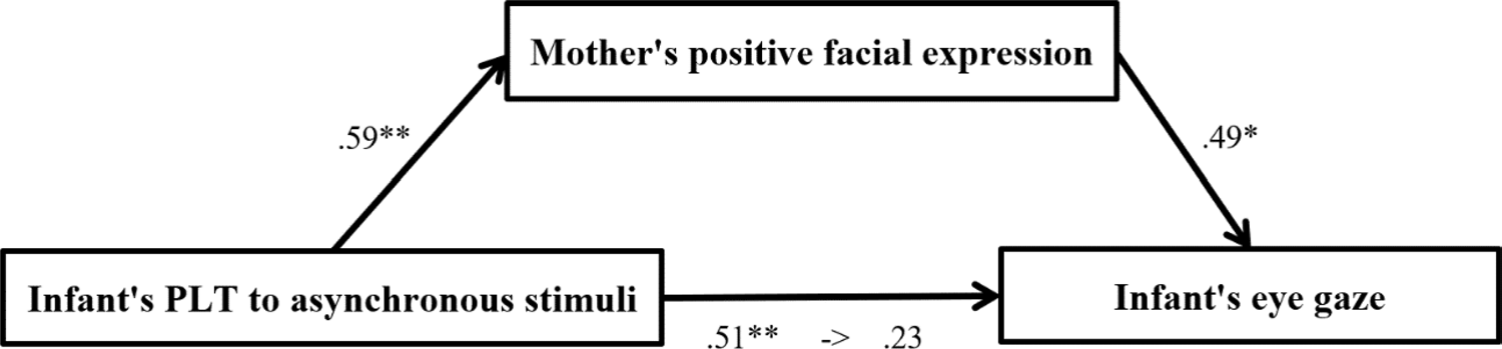
Mediation analysis: PLT toward the cardio-visual asynchronous stimuli in infants, mother’s positive facial expression, and infant’s eye gaze toward mother’s face.

Based on the correlation analysis, we found that the criteria for mediation analysis were met in the relationship between PLT toward the cardio-visual asynchronous stimuli in infants, the mother’s positive facial expression, and the infant’s eye gaze toward the mother’s face (Fig. 3). In the mediation model, the PLT toward the cardio-visual asynchronous stimuli in infants was entered as the independent variable, the mother’s positive facial expression was entered as the mediator, and the infant’s gaze toward the mother’s face was entered as the dependent variable. The confidence intervals were based on 2000 bootstrap samples. PLT toward the cardio-visual asynchronous stimuli in infants was positively associated with mothers’ positive facial expressions (*B* = 0.586, *t*(23) = 3.46, *p* = 0.002). Mothers’ positive facial expressions were positively associated with the infant’s eye gaze toward the mother (*B* = 0.486, *t*(22) = 2.42, *p* = 0.024). PLT toward the cardio-visual asynchronous stimuli in infants was positively associated with infants gaze toward the mother’s face (*B* = 0.514, *t*(23) = 2.87, *p* = 0.009). Results of the mediation analysis using the bootstrapping method confirmed the mediating role of mother’s positive facial expression in the relationship between PLT toward the cardio-visual asynchronous stimuli in infants and infants’ eye gaze toward the mother’s face (95% confidence interval [CI] 0.287–3.482). In addition, the direct effect of PLT toward cardio-visual asynchronous stimuli in infants on infants gaze toward the mother’s face became non-significant (*B* = 0.229,* t*(22) = 1.144, *p* = 0.265) when controlling for mothers’ positive facial expressions, suggesting full mediation and explaining 51.4% of the relationship.

**Figure 4:**
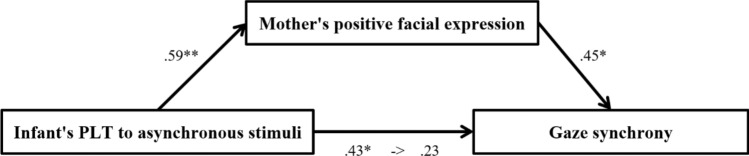
Mediation analysis: PLT toward the cardio-visual asynchronous stimuli in infants, mother’s positive facial expression, and gaze synchrony.

In another mediation model, as shown in Fig. 4, PLT toward cardio-visual asynchronous stimuli in infants was positively associated with mothers’ positive facial expressions (*B* = 0.586, *t*(23) = 3.46, *p* = 0.002). Mothers’ positive facial expressions were positively associated with gaze synchrony (*B* = 0.452, *t*(22) = 2.23, *p* = 0.036). PLT toward the cardio-visual asynchronous stimuli in infants was positively associated with gaze synchrony (*B* = 0.519, *t*(23) = 2.91, *p* = 0.008). We found mediating effects of mothers’ positive facial expressions on the relationship between PLT toward cardio-visual asynchronous stimuli in infants and gaze synchrony (95% confidence interval [CI] 0.203–3.359). The direct effect of PLT toward cardio-visual asynchronous stimuli in gaze synchrony became non-significant (*B* = 0.254,* t*(22) = 1.25, *p* = 0.224) when controlling for mothers’ positive facial expressions, suggesting full mediation and explaining 51.9% of the relationship. No mediation effect was found in the relationships between infants’ PLT and asynchronous stimuli, mothers’ positive facial expression, and affect synchrony.

## Discussion

The present study examined whether and how infants’ and mothers’ interoception is associated with social behaviors in infant-mother interactions. Our main results are as follows: (1) Infants showed a significant visual preference for cardio-visual asynchronous stimuli, while mothers showed a significant visual preference for cardio-visual synchronous stimuli; (2) infants’ preference for cardio-visual asynchronous stimuli was positively correlated with their eye contact and gaze/affect synchrony, and mothers’ preference for cardio-visual synchronous stimuli was positively correlated with infants’ eye contact and gaze/affect synchrony; and (3) infants with higher interoception received more smiles from their mothers and made more eye contact with their mothers during social interactions.

We found that infants were able to detect cardio-visual synchrony. This is consistent with a previous study that showed that infants prefer cardio-visual asynchronous stimuli to synchronous stimuli^14^. The results of this study also showed that mothers could detect cardio-visual synchrony, although their preference for synchrony was opposite to that of the infants. The difference in the direction of infant’s and mother’s preferences for cardio-visual synchrony may be due to the fact that children tend to prefer novel stimuli, while adults tend to prefer familiar stimuli^20–22^. The preference for cardio-visual synchrony, together with familiarity/novelty response, can also be considered a more active explanation in terms of top-down attentional processes^20–22^. That is, infants showed a preference for asynchronous cardio-vascular stimuli, which may be due to a greater allocation of attention to the discrepancy between visual stimuli and the sensation of one's heartbeat. However, because mothers have well-developed attentional functions related to the sensation of their own heartbeat, they pay more attention to stimuli that match their sense of their own heartbeat to visual stimuli. In summary, the behavioral measures using the preferential looking paradigm in this study allowed us to assess the sensitivity of infants and mothers to interoceptive signals.

Interoception is related to social cognition (i.e., understanding others such as empathy and mentalizing) in adults^4,6–8,11,25,26^. Integrating interoceptive signals with exteroception are thought to be associated with the attachment of value to the environment. This can lead to the development of social cognition. Therefore, if interoception and social behavior are correlated, infants who are sensitive to interoception are more likely to perceive changes in body signals caused by interactions with others, and are more likely to learn the value of social cues. For example, if infants experience more smiles from a caregiver, those who are sensitive to interoception may acquire the internal model of the caregiver as a reward^13^ and engage in more social behaviors.

The present findings indicate that infants who preferred cardio-visual asynchronous stimuli showed more eye contact and gaze/affect synchrony. If interoception is important for learning social motivation, this correlation suggests that the integration of interception and exteroception may play a role in social behavior. Mediation analysis showed that infants with a higher preference for cardio-visual asynchronous stimuli made more eye contact through more smiles from their mothers. These results suggest that the infant's sensitivity to cardiac signals plays a role in the infant-mother interaction in relation to the process of valuing the smile of others, ultimately increasing sensitivity to social cues, such as the mother’s gaze.

The present study also showed that mothers preferred cardio-visual synchronous stimuli, suggesting that the preferential looking method can be used to assess interoception in adults. However, the validity of the present task needs to be examined in the future because the relationship between the evaluation of internal receptive sensation by the preference gaze method and the evaluation of existing tasks (e.g., heartbeat counting task^27^) has not been examined. Furthermore, there was no association between mothers’ interoception and their social behavior toward infants. In the 3-min infant–mother interaction observed in the present study, many mothers behaved well socially, which may be a ceiling effect. This may have prevented us from identifying these relationships. In the future, it will be necessary to examine longer-term infant–mother interactions and verify their relationship with interoception.

Finally, we discuss several limitations and future considerations for investigating the contribution of interoception to sensitivity to social cues in the current study. Although we found that infants’ interoception was associated with eye contact behavior in infant–mother interactions, mediated by the mother's smile, it is difficult to identify causal relationships between variables in this study because we assessed interoception and social behavior separately at the same time. For example, a longitudinal study could examine whether interoception predicts individual differences in later social behavior, or an experimental study could examine whether social cues affect interoception. The possible influence of interoception on social cognitive development should be examined in future studies. In addition, while we were able to obtain significant full mediation effects in the sample size^28^, the sample size of 25 was relatively small for examining mediation effects^29^. Future studies with larger sample sizes are needed to address this issue. Moreover, we assessed interoception by measuring the cardio-visual preference task using the preferential looking paradigm. In a previous study, infants’ interoception was assessed using an electrophysiological index of cortical cardiac processing (heartbeat evoked potential [HEP]) in addition to a similar behavioral index^14^. In future studies, a combination of multiple measures to assess interoception will allow us to further examine the developmental principles of social behavior.

In conclusion, while researchers have long been interested in the role of interoception in social cognitive development, there is an absence of evidence on how interoception is related to social cognition in infants. Our findings demonstrated that infants with higher interoception made more eye contact with their mothers, mediated by mothers’ smiling. Based on the hypothesis that interoception plays a role in sensitivity to social cues^13^, these findings suggest that infants' high sensitivity to interception may contribute to their perception of others' smiles as a reward, and consequently promote eye contact behavior with the mother. The present study revealed that in infants, as in adults^4–9^, interoception is associated with sensitivity to social cues, such as smiles and gaze.

## Supplementary Information

Supplementary Information.

## Data Availability

All data analyzed during this study are available upon reasonable request.
