# Supplementary material for: Infants’ interoception is associated with eye contact in dyadic social interactions
Source: Sci Rep. 2023 Jun 19;13:9520. doi: 10.1038/s41598-023-35851-9 (PMC10279682; doi:10.1038/s41598-023-35851-9)
Supplement: Supplementary file 1 — Supplementary Information. [file 41598_2023_35851_MOESM1_ESM.docx]

**Infants’ interoception is associated with eye contact in dyadic social interactions**

Masahiro Imafuku*, Hiromasa Yoshimoto, Kazuo Hiraki

**Appendix A: Kappa coefficients (κ) for coding of social behaviors in infant–mother interactions.**

Table S1: Kappa coefficients (κ) for coding.

**
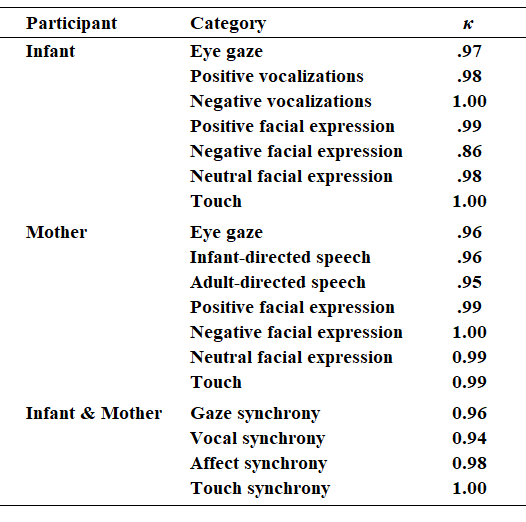
**
